# Supplementary material for: Ameliorative effects of Penthorum chinense Pursh on insulin resistance and oxidative stress in diabetic obesity db/db mice
Source: PLoS One. 2024 Oct 7;19(10):e0311502. doi: 10.1371/journal.pone.0311502 (PMC11458015; doi:10.1371/journal.pone.0311502)
Supplement: S1 Raw data — (PDF) [file pone.0311502.s001.pdf]

Supplementary Table 1. Raw data

	NC	MC	LPCP	MPCP	HPCP
**FBG (mmol/L)**					
0	4.61±0.72	20.03±1.92	8.46±1.19	18.73±1.39	19.70±2.60
2 w	4.11±0.86	23.16±3.06	18.16±1.74	18.44±5.35	17.13±3.96
4 w	3.29±0.65	24.53±5.83	16.33±3.49	16.10±4.01	14.54±2.07
**HbA1c (ng/mL)**					
	2.13±0.22	11.60±2.75	9.37±1.96	8.04±1.52	7.46±1.60
**OGTT (mmol/L)**					
0	5.98±1.16	28.07±4.00	25.73±2.87	25.24±4.09	23.63±3.08
30 min	7.80±1.33	37.23±3.05	36.10±3.37	35.94±4.30	34.40±3.54
60 min	6.83±1.00	37.00±3.07	34.47±4.25	34.14±4.72	29.43±5.30
120 min	4.60±0.76	32.04±3.57	26.79±3.17	26.66±1.82	24.87±1.88
**AUC of OGTT (mmol/L. h)**					
	12.13±1.55	67.06±3.80	50.23±4.17	60.73±5.97	60.09±5.12
**Insulin (mIU/L)**					
	4.77±1.66	12.30±3.23	11.02±2.57	9.03±2.72	7.48±2.20
**HOMA-IR**					
	0.69±0.28	13.65±5.84	7.87±2.20	6.54±2.98	4.88±1.82
**Food intake (g)**					
1 w	23.33±3.06	56.67±4.16	49.67±6.25	46.50±3.50	43.17±9.78
2 w	22.57±5.43	64.41±3.41	62.03±8.15	47.75±2.48	45.69±1.42
3 w	22.98±6.49	64.91±4.28	56.73±1.88	53.78±6.21	52.11±9.09
4 w	19.30±2.29	59.86±2.80	55.47±5.85	48.39±3.16	45.64±5.61
**Body weights (g)**					
0	23.58±0.98	43.91±1.90	43.48±1.26	43.58±1.79	43.63±1.69
1 w	23.86±1.07	44.40±2.25	43.00±1.15	43.25±1.28	42.01±2.68
2 w	24.34±0.57	44.71±2.75	42.66±1.49	40.75±0.89	40.46±3.02
3 w	24.83±0.83	45.89±2.74	42.31±2.08	40.20±3.05	38.75±2.05
4 w	24.96±0.68	46.09±2.65	41.63±2.62	39.45±2.77	38.73±1.89
**TG (mmol/L)**					
	1.47±0.27	2.77±0.08	2.53±0.22	2.47±0.18	2.39±0.17
**TC (mmol/L)**					
	2.35±0.74	4.23±0.84	3.76±0.77	3.88±0.79	3.32±0.48
**LDL-C (mmol/L)**					
	2.14±0.34	4.16±0.36	3.69±0.05	3.82±0.17	3.08±0.51
**HDL-C (mmol/L)**					
	1.76±0.05	1.14±0.11	1.22±0.27	1.33±0.13	1.40±0.22
**TG/HDL-C**					
	0.84±0.17	1.91±0.19	2.14±0.42	1.87±0.15	1.74±0.25
**TC/HDL-C**					
	1.33±0.42	3.76±0.96	3.13±0.66	2.95±0.68	2.41±0.48
**LDL-C/HDL-C**					
	1.21±0.17	3.67±0.4	3.13±0.61	2.9±0.39	2.24±0.47
**SOD (U/mgprot)**					
	352.53±10.17	284.46±24.30	296.38±21.21	316.90±18.44	321.89±16.69
**GSH-Px (U/mgprot)**					

	268.45±10.47	225.68±4.74	230.89±9.94	233.15±12.73	238.49±12.54
**MDA (mmol/mgprot)**	0.22±0.03	0.48±0.08	0.40±0.09	0.36±0.08	0.34±0.07
