# Supplementary material for: Ameliorative effects of Penthorum chinense Pursh on insulin resistance and oxidative stress in diabetic obesity db/db mice
Source: PLoS One. 2024 Oct 7;19(10):e0311502. doi: 10.1371/journal.pone.0311502 (PMC11458015; doi:10.1371/journal.pone.0311502)
Supplement: S1 Fig — (PDF) [file pone.0311502.s002.pdf]

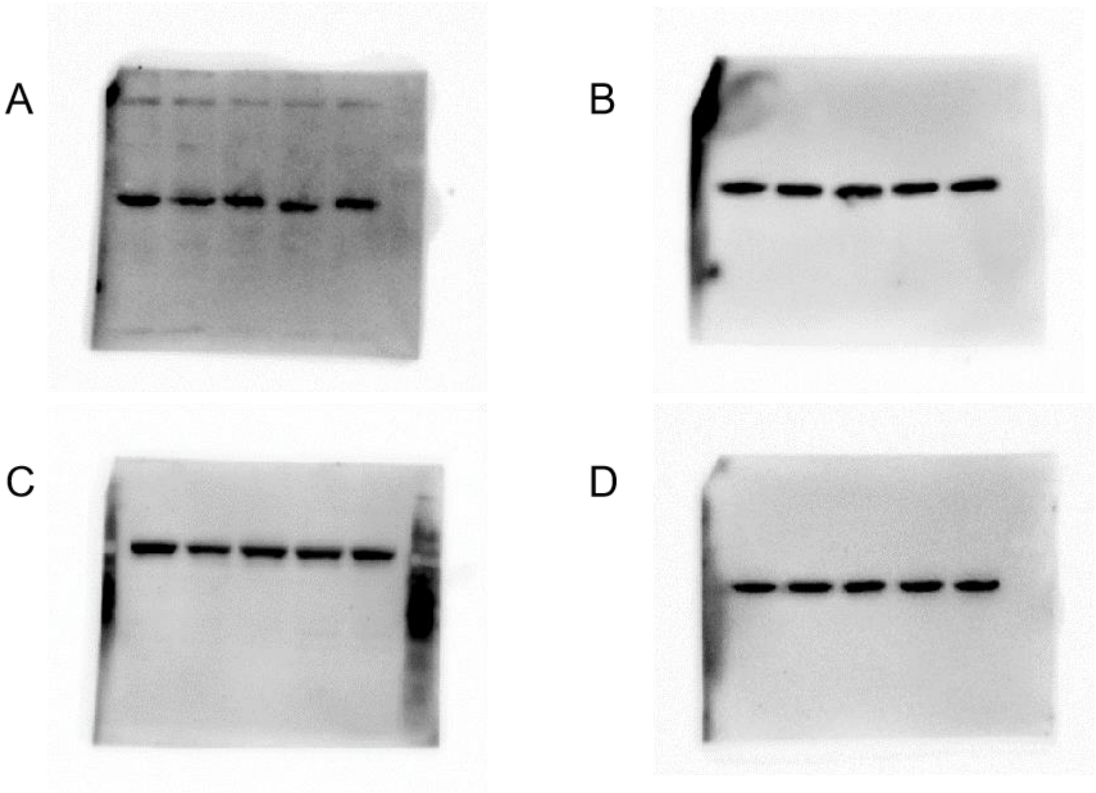

Supplementary Figure 2. Original image of western blot analysis. (A) GLUT2; (B) β-actin of GLUT2; (C) GCK; (D) β-actin of GCK.
