# Supplementary material for: Ameliorative effects of Penthorum chinense Pursh on insulin resistance and oxidative stress in diabetic obesity db/db mice
Source: PLoS One. 2024 Oct 7;19(10):e0311502. doi: 10.1371/journal.pone.0311502 (PMC11458015; doi:10.1371/journal.pone.0311502)
Supplement: Attachment — Submitted filename: Accept after minor revision.docx [file pone.0311502.s003.docx]

Accept after minor revision.

Comments already uploaded

1. Language should be improved and spelling mistakes should be corrected.

2. Please abbreviate the text at first instance.

3. Give 2-3 lines about current status of diabetes at global level.

Brauer, Michael, Roth, Gregory A., Aravkin, Aleksandr Y., Zheng, Peng, Abate, Kalkidan Hassen, Abate, Yohannes Habtegiorgis, Abbafati, Cristiana, Kavita Munjal et al., 2024. Global burden and strength of evidence for 88 risk factors in 204 countries and 811 subnational locations, 1990–2021: a systematic analysis for the Global Burden of Disease Study 2021. The Lancet. 403, 2162-2203. https://doi.org/10.1016/S0140-6736 (24)00933-4.

Gauttam, V., Munjal, K., Mujwar, S., Sawale, J., Rohilla, M., Gupta, S. 2022. Comparative Study of Developed Formulation and Market Formulation for Antidiabetic Potential in Alloxan Induced Diabetic Wistar Rats. Journal of Young Pharmacists. 2022; 14(4), 387-393.
