# Supplementary material for: Ameliorative effects of Penthorum chinense Pursh on insulin resistance and oxidative stress in diabetic obesity db/db mice
Source: PLoS One. 2024 Oct 7;19(10):e0311502. doi: 10.1371/journal.pone.0311502 (PMC11458015; doi:10.1371/journal.pone.0311502)
Supplement: Attachment — Submitted filename: Point-by-point response to reviewers and editors.docx [file pone.0311502.s004.docx]

Dear Editor and Reviewers,

We sincerely thank you for your valuable feedback which helped to improve the quality of our manuscript entitled “Ameliorative Effects of *Penthorum chinense Pursh* on Insulin Resistance and Oxidative Stress in diabetic obesity *db/db* mice” (Manuscript ID: PONE-D-24-18781). We have considered your comments word by word and have made revisions based on your suggestions question by question. The editor's and reviewers’ comments are listed in blue italics and specific concerns have been numbered. Our response is given in normal font and changes/additions to the manuscript are marked in red. The main amendments in the paper and the responses to your comments are listed as follows:

**For reviewer 1#**

*1. A few grammatical and typographical errors were noted. For instance, ANIMALS EXPERIMENT, instead of ANIMAL EXPERIMENTS.*

Response: Thank you for your meticulousness. We felt sorry about this low-grade mistake and the amendment is in red.

*2. De Ritis ratio and Ferritin could have been included.*

Response: Thank you for your advice. *Penthorum chinense* Pursh (PCP) is widely utilized in China to treat a variety of liver diseases. Previous studies have demonstrated that the levels of aspartate aminotransferase (AST) and alanine aminotransferase (ALT) were ameliorated in a dose-dependent manner by PCP [1-4]. Therefore, we did not measure liver function-related indicators in this study and unfortunately, we were unable to calculate the De Ritis ratio either. As for ferritin, there is no published literature report on the effect of PCP on it. However, our team's ongoing but unpublished study suggests that total flavonoids of PCP were able to significantly attenuate liver injury of iron-overloaded rats by up-regulating the expression of divalent metal ion transporter protein 1 and transferrin receptor 1. Of course, if we can get new funds, we shall carry out more experiments to dig into the effect of PCP on ferritin.

*3. The authors are advised to compute lipid ratios, because of their link with both diabetes mellitus and steatosis.*

Response: Thank you for your advice. T2DM patients often present with characteristic plasma lipid and lipoprotein abnormalities, including low HDL-C, high LDL-C, and elevated TC and TG levels [5], which is consistent with our findings. Considering the reviewer’s suggestion, we further analyzed the lipid ratio parameters, including TC/HDL-C, TG/HDL-C, and LDL-C/HDL-C, that considered could predict the risk of T2DM complications by prior studies [6], especially for coronary heart disease. The results of lipid ratio parameters are shown in Fig 2G-I. Those in the NC, including TC/HDL-C, TG/HDL-C, and LDL-C/HDL-C were significantly higher than those in the *db/db* mice (*P*<0.01). The values of TG/HDL-C in the MPCP and HPCP were significantly lower than those in the MC (*P*<0.05), while those of LPCP were not significantly different from those of the MC. However, the values of TG/HDL-C in the LPCP were significantly higher than those in the HPCP (*P*<0.05). All doses of PCP treatment decreased the values of LDL-C/HDL-C in a dose-response relationship (*P*<0.05), demonstrated by significantly lower values of LDL-C/HDL-C in the HPCP than those in the LPCP and MPCP (*P*<0.01).

Fig 2. Effects of PCP on (A) food intake and (B) body weights, and serum lipid levels of (C) TG, (D) TC, (E) LDL-C, (F) HDL-C, (G) TG/HDL, (H) TC/HDL-C and (I) LDL-C/HDL-C in *db/db* mice. Data are expressed as mean ± SD; n = 8 for each experimental group. **P*<0.05, ***P*<0.01 vs. normal control group. ^#^*P*<0.05, ^##^*P*<0.01 vs. model control group. ^a^*P*<0.05, ^aa^*P*<0.01 vs. low dose of PCP. ^b^*P*<0.05, ^bb^*P*<0.01 vs. medium dose of PCP. ^c^*P*<0.05, ^cc^*P*<0.01 vs. high dose of PCP.

*4. Was the ELISA kit for HbA1c, a high-sensitivity and specificity one with minimal cross-reactivity? Why did the authors not use HPLC?*

Response: Thank you for your question. According to the literature, various methods developed for HbA1c examination include immunoassay, boronated affinity, enzymatic, capillary electrophoresis, and Ion-Exchange High-Performance Liquid Chromatography (IE-HPLC) [7], and each has its advantages and limitations. Currently, the two most used methods for HbA1c detection are IE-HPLC and immunoassay, and the results of them are in strong agreement in most cases. In addition to its advantages in detecting HbA1c, IE-HPLC methods can also detect other types of hemoglobin and the presence of hemoglobin variants, which are widely used in clinical practice. However, we did not choose IE-HPLC due to its high price. ELISA kits working on the principle of immunoassay, are one of the methods with high sensitivity, stable efficacy, and low price for the determination of HbA1c, which are widely used in animal experiments [8]. In summary, we finally chose the ELISA kits to measure the HbA1c levels of *db/db* mice.

**For reviewer 2#:**

*1. Language should be improved and spelling mistakes should be corrected.*

Response: Thank you for your suggestion. We have invited a native English speaker to help polish our manuscript, and we hope the revised manuscript will be acceptable to you.

*2. Please abbreviate the text at the first instance.*

Response: Thank you for your suggestion. We felt sorry about this low-grade mistake and now all full names with their abbreviations have been added when they first appear. The amendment is in red.

*3. Give 2-3 lines about* *the status of diabetes at the global level.*

Response: Thank you for providing valuable references. We have checked the literature carefully and added more description about the status of diabetes at the global level in the INTRODUCTION part of the revised manuscript (Lines 57-60).

**For reviewer 3#:**

*1. The inter-group significance shown in the picture is inconsistent with the description in the text. For example, in the result section ‘PCP improved parameters related to hyperglycemia and IR in db/db mice’ (Line 186), according to the figure, it should be ‘P<0.01’.*

Response: Thank you for your meticulousness. We felt sorry about this low-grade mistake and the amendment is in red.

*2. In Fig5B,* *the significance may be wrong, especially HPCP Group. Please check through the manuscript to avoid these errors.*

Response: Thank you for your meticulousness. We have checked and reanalyzed the raw data and unfortunately, the results and significance were real. As shown in Fig 5B, when compared to the MC, the relative expression of GCK protein increased in the LPCP, HPCP (*P* < 0.05), and MPCP (*P* < 0.01), respectively. However, there was no significant difference in multiple comparisons between the three PCP-treatment groups. We considered the following two possible reasons. Firstly, a smaller p-value does not mean that the difference is larger, only that more reason to think that there is a difference between the two groups. In this study, the relative expression of GCK protein in the MPCP and HPCP was significantly higher than the MC, but the p-value was smaller in the MPCP, which only means there was more reason to think that the MPCP is different from the MC. Secondly, the above results may be due to the relatively small sample size of the experiment.

**For Journal Requirements:**

*1. Please ensure that your manuscript meets* *PLOS ONE's style requirements, including those for file naming.*

Response: Thanks for the heads up. We have revised the manuscript against PLOS ONE's style requirements.

*2. To comply with PLOS ONE submissions requirements, in your Methods section, please provide additional information regarding the experiments involving animals and ensure you have included details on (1) methods of sacrifice, and (2) efforts to alleviate suffering.*

Response: Thank you for your meticulousness. We have added information related to animal sacrifice in the section on Materials and methods (Lines 43-46).

*3.* *We note that the grant information you provided in the ‘Funding Information’ and ‘Financial Disclosure’ sections does not match. When you resubmit, please ensure that you provide the correct grant numbers for the awards you received for your study in the ‘Funding Information’ section.*

Response: Thanks for the meticulousness. However, no item for 'Funding Information' was found in the revising system. We have added the “Financial Disclosure” in the Cover Letter and Revised Manuscript.

*4. In the online submission form, you indicated that all data generated or analyzed during this study are included in this published article. The raw data are available from the corresponding author on reasonable request. All PLOS journals now require all data underlying the findings described in their manuscript to be freely available to other researchers, either 1. In a public repository, 2. Within the manuscript itself, or 3. Uploaded as supplementary information. This policy applies to all data except where public deposition would breach compliance with the protocol approved by your research ethics board. If your data cannot be made publicly available for ethical or legal reasons (e.g., public availability would compromise patient privacy), please explain your reasons for resubmission and your exemption request will be escalated for approval.*

Response: Thanks for the heads up. We have uploaded the raw data as supplementary information.

*5. Your ethics statement should only appear in the Methods section of your manuscript. If your ethics statement is written in any section besides the Methods, please delete it from any other section.*

Response: Thanks for the heads up. We have modified it as required.

6. *PLOS ONE now requires that authors provide the original uncropped and unadjusted images underlying all blot or gel results reported in a submission’s figures or Supporting Information files. This policy and the journal’s other requirements for blot/gel reporting and figure preparation are described in detail at* [*https://journals.plos.org/plosone/s/figures#loc-blot-and-gel-reporting-requirements*](https://journals.plos.org/plosone/s/figures#loc-blot-and-gel-reporting-requirements) *and https://journals.plos.org/plosone/s/figures#loc-preparing-figures-from-image-files. When you submit your revised manuscript, please ensure that your figures adhere fully to these guidelines and provide the original underlying images for all blot or gel data reported in your submission. See the following link for instructions on providing the original image data: https://journals.plos.org/plosone/s/figures#loc-original-images-for-blots-and-gels.  In your cover letter, please note whether your blot/gel image data are in Supporting Information or posted at a public data repository, provide the repository URL if relevant, and provide specific details as to which raw blot/gel images, if any, are not available. Email us at* [*plosone@plos.org*](mailto:plosone@plos.org) *if you have any questions.*

Response: Thanks for the heads up. We have uploaded the raw blot/gel images as supplementary information.

7. *Please review your reference list to ensure that it is complete and correct. If you have cited papers that have been retracted, please include the rationale for doing so in the manuscript text, or remove these references and replace them with relevant current references. Any changes to the reference list should be mentioned in the rebuttal letter that accompanies your revised manuscript. If you need to cite a retracted article, indicate the article’s retracted status in the References list and include a citation and full reference for the retraction notice.*

Response: Thanks for the heads up. We have checked the reference list and ensure it is complete and correct.

**Other changes:**

1. The layout of **Fig 1** has been changed from 2 x 3 to 3 x 2 for better aesthetics and uniformity in the revised manuscript.

2. According to the reviewer’s suggestion, **Fig 2G-I** has been added to **Fig 2**, and the layout of **Fig 2** has been replaced from 2×3 to 3×3.

3. Changes in vocabulary and grammar have been revised in red.

Finally, we would like to express our thanks again for your reviews and guidance on our manuscript. We sincerely hope that with the help of your knowledge, we can complete an excellent paper and we also hope our paper can be published in your journal.

Thank you and best regards.

Yours Sincerely,

Ning Lin

Clinical Nutrition, The General Hospital of Western Theater Command, Sichuan, Chengdu 610083, China.

Email: helenmedic@yeah.net

July 31, 2024

**Reference**

1.	Du YC, Lai L, Zhang H, Zhong FR, Cheng HL, Qian BL, et al. Kaempferol from Penthorum chinense Pursh suppresses HMGB1/TLR4/NF-κB signaling and NLRP3 inflammasome activation in acetaminophen-induced hepatotoxicity. Food & function. 2020;11(9):7925-34.

2.	Jiang Y, Zhong M, Zhan H, Tao X, Zhang Y, Mao J, et al. Integrated strategy of network pharmacology, molecular docking, HPLC-DAD and mice model for exploring active ingredients and pharmacological mechanisms of Penthorum chinense Pursh against alcoholic liver injury. Journal of ethnopharmacology. 2022;298:115589.

3.	Wang S, Li W, Liu W, Yu L, Peng F, Qin J, et al. Total flavonoids extracted from Penthorum chinense Pursh mitigates CCl(4)-induced hepatic fibrosis in rats via inactivation of TLR4-MyD88-mediated NF-κB pathways and regulation of liver metabolism. Frontiers in pharmacology. 2023;14:1253013.

4.	Zhang H, Cui X, Liu W, Xiang Z, Ye JF. Regulation of intestinal microflora and metabolites of Penthorum chinense Pursh on alcoholic liver disease. Frontiers in pharmacology. 2023;14:1331956.

5.	Bahiru E, Hsiao R, Phillipson D, Watson KE. Mechanisms and Treatment of Dyslipidemia in Diabetes. Current cardiology reports. 2021;23(4):26.

6.	Gu X, Yang X, Li Y, Cao J, Li J, Liu X, et al. Usefulness of Low-Density Lipoprotein Cholesterol and Non-High-Density Lipoprotein Cholesterol as Predictors of Cardiovascular Disease in Chinese. The American journal of cardiology. 2015;116(7):1063-70.

7.	Rhea JM, Molinaro R. Pathology consultation on HbA(1c) methods and interferences. American journal of clinical pathology. 2014;141(1):5-16.

8.	Huang D, Jiang Y, Chen W, Yao F, Huang G, Sun L. Evaluation of hypoglycemic effects of polyphenols and extracts from Penthorum chinense. Journal of ethnopharmacology. 2015;163:256-63.
